# Accuracy and Robustness of Bone Volume Fraction Assessment by Photon-Counting, Dual-Energy, and Quantitative CT using Micro-CT as Standard of Reference

**DOI:** 10.1007/s10439-025-03732-z

**Published:** 2025-06-11

**Authors:** Patrik Wili, Dominic Gascho, Alice Dudle, Roy P. Marcus, Daniel Nanz, Michael Thali, Philippe Zysset

**Affiliations:** 1 Zurich Institute of Forensic Medicine, University of Zurich, Zurich, Switzerland; 2 ARTORG Centre for Biomedical Engineering Research, University of Bern, Bern, Switzerland; 3 Department of Radiology, Balgrist University Hospital, University of Zurich, Zurich, Switzerland; 4 Swiss Center for Musculoskeletal Imaging, Balgrist Campus AG, Zurich, Switzerland; 5 Medical Faculty, University of Zurich, Zurich, Switzerland

**Keywords:** Quantitative CT, Dual-energy CT, Photon counting CT, Virtual monochromatic images, Bone density

## Abstract

**Purpose:**

We aimed to quantitatively compare different computed tomography (CT)-based bone volume fraction (BV/TV) measurements. We hypothesize that phantom-less measurement using virtual monochromatic images (VMI) reconstructed form dual-energy CT (DECT) or photon counting CT (PCT) is less affected by tissue variations in trabecular bone than quantitative CT (QCT) and that PCT allows measurements with a lower radiation dose.

**Method:**

The BV/TV of bovine trabecular bone samples were measured using four CT scanning methods. Eight different measurements were compared using three soft tissue substitutes (air, saline solution, and fat) to investigate its effect on BV/TV estimation. For this purpose, the samples’ bone marrow was removed and replaced with one of the three substitutes in succession for CT scanning. For QCT, a standard bone phantom was used to derive BV/TV form CT values. While DECT- and PCT-based measurements were based on a system of energy-dependent equations established by reconstructing VMIs for specific photon energies. *T*-test, ANOVA tests and pairwise comparison were performed using the micro-CT (µCT) measurements as reference standard.

**Results:**

QCT showed a significant difference between the fat, saline solution, and air. DECT and especially PCT showed no differences between the substitutes. PCT showed no significant differences between radiation doses.

**Conclusion:**

Our results highlight the complexity of BV/TV measurements and emphasize the impact of the trabecular bone components on measurement accuracy. Despite these challenges, VMIs from “low” dose PCT provide a reliable alternative to standard QCT. They have the potential to improve the estimation of bone conditions, as well offering valuable insights for clinical, and forensic applications.

## Introduction

Nowadays, various X-ray-based methods are available to characterize or measure bone density at multiple skeletal sites. For example, two-dimensional dual-energy X-ray absorptiometry (DEXA)-based areal bone mineral density (aBMD) measurement can be performed at the femoral neck and lumbar spine and is the current clinical standard of care for screening and diagnosing osteoporosis [1, 2]. The two main limitations of DEXA are the inability to differentiate between cortical and trabecular bone and the lack of 3D information [1, 3, 4]. Advanced medical imaging based on computer tomography (CT) using X-ray absorption produces three-dimensional images of the skeletal structure. The three-dimensional nature of CT images enables the separate assessment of cortical and trabecular bone [5, 6]. Beyond the morphological characteristics of bone, it is of interest to quantify volumetric density measures such as the volumetric bone mineral density (vBMD) or the bone volume fraction (BV/TV), as indicated by the bone volume (BV) to total volume (TV) ratio. Indeed, these volumetric measures facilitate the exploration of diagnostic inquiries extending beyond osteoporosis [6], especially the prediction of bone’s elastic and yield properties [7–10]. The current clinical standards to measure vBMD, which is related to BV/TV, is a quantitative CT (QCT) [7]. vBMD-based BV/TV is derived from QCT assuming constant tissue mineralization [7, 11, 12], which has been shown as a reasonable assumption [13].

QCT-based vBMD measurement either requires a calibration phantom to be placed inside the scan field during image acquisition or utilizes asynchronous calibration. These phantoms feature inserts with varying concentrations of hydroxyapatite (HA). This enables the mapping of voxel-specific attenuation information, expressed in Hounsfield units (HU), to equivalent densities of HA by leveraging the known densities of the phantom inserts [9, 14]. A significant limitation of QCT lies in the simplified assumption that variations in the bone’s X-ray attenuation are solely attributable to changes in bone mineralization. This perspective is manifested through the composition of the phantoms, which only contain hydroxyapatite and a surrogate for water, neglecting the impact of variations in soft tissues occupying the intertrabecular space, such as fat or red bone marrow [14–16]. This assumption is partially valid because within trabecular bone, the mineralized tissue significantly contributes to X-ray attenuation per unit volume. Nevertheless, age-related increases in fat concentration within bone marrow, situated in the intertrabecular space, alter CT attenuation, potentially affecting the derived vBMD values [14, 15]. Fat has a lower X-ray attenuation coefficient compared to water. Thus, increased fat content in the marrow can lead to an underestimation of QCT-based vBMD [17]. Additionally, bleeding and/or increased fluid accumulation in the marrow could cause local elevation of the attenuation coefficient, resulting in an overestimation of vBMD [14]. Furthermore, the presence of a calibration phantom can increase the radiation dose (CTDI_v_, standardized measure for radiation dose output), to the patient, due to the increase of the current applied by the CT protocols to maintain contrast [2]. Moreover, the position of the calibration phantom can influence the accuracy of QCT-based vBMD measurement [9]. Lastly, the appearance of small gas impurities during preclinical *in vitro* tests on extracted bone samples or post-mortem measurements could lead to a reduction in the vBMD values. This is due to the almost un-attenuated transmission of X-rays through air.

In contrast, spectral CT-based BV/TV measurement, a phantom-less and a relatively unexplored method for direct BV/TV measurement involves the use of virtual monochromatic images (VMI) obtained from spectral CT technologies, including dual-energy CT (DECT) and photon counting CT (PCT) [18]. There the energy dependence of the photoelectric effect at different X-ray spectra is used for direct quantification of BV/TV [3, 11]. It allows the reconstruction of VMIs, material decomposition by combining the information from measurements at different photon energies, and provides the opportunity to reduce the effects of variations in soft tissue composition [11, 14, 19] and the radiation dose. The concept of using different X-ray spectra to decompose trabecular bone into its components is not new. Already in 1988, Nickoloff et al. [11] developed a model which uses the information of two different X-ray spectra to differentiate trabecular tissue into its two main macro structural components: the bone extracellular matrix (ECM; mineral and organic) and marrow (fat, red marrow and water). Their model assumes that the average density of the bone ECM remains constant, that water and red bone marrow are similar enough in their X-ray attenuation properties to combine the two in non-adipose tissue and that the photon-electric effect of one spectrum is dominated by the mean photon energy of the spectra. The problem of the last assumption is that the X-ray spectra are not known and change over time. Nevertheless, based on this method, multiple *in vitro* and *in vivo* studies have shown promising results for a more accurate vBMD assessment using DECT which may allow for opportunistic vBMD screening [3, 14, 17, 19, 20] in clinical settings. Among these, a recent HA-phantom study highlighted higher diagnostic accuracy of DECT-based bone density compared to QCT [3]. Contrary to DECT, PCT is based on a new detector technology and enables substantial CTDI_v_ reduction without deterioration of performance levels in the assessment of bone mineral density [21]. Furthermore, PCT does not require a specialized scanning sequence for acquiring spectral data, unlike most DECT [22] scanners.

A main limitation of these systems is the low uptake of PCT systems in clinical practice. While DECT systems are more prevalent, there is often a lack of experienced operators or multidisciplinary engagement. Despite these challenges, both DECT and PCT systems are becoming increasingly available for research purposes. Therefore, and because of the possibility of phantom-less calibration, they have the potential to reduce the burden to gather data for preclinical and clinical studies relying on measurements from routine radiological practice.

Additionally, in an experimental context, highly precise micro-CT (µCT) images, which are not suitable for clinical application, are used as a reference to measure BV/TV [7, 23, 24], especially for biomechanical analysis [4]. All these techniques build up on the same principle which relates the amount of X-ray attenuation to main bone constituents [11].

To the best of our knowledge, the impact of soft tissue alterations on BV/TV measurements using spectral images derived from DECT and PCT has not yet been directly compared to QCT using real bone samples, with high-resolution µCT-based BV/TV values serving as a reference. So far, spectral-based BV/TV measurements have used the reconstructed images from the two different X-ray spectra for material dissection and not VMIs. We hypothesize that; (1) phantom-less VMI-based BV/TV measurements using DECT and PCT is less sensitive to alterations in soft tissue within trabecular bone compared to QCT, thereby enabling a more precise assessment of BV/TV; (2) accuracy of PCT-based BV/TV measurements are CTDI_v_-independent and allow more dose-efficient measurements; (3) PCT and DECT demonstrate greater robustness against air or gas occlusions compared to QCT. Thus, the use of virtual monochromatic images (VMIs) for BV/TV measurement generated by spectral CT technologies, namely DECT and PCT, offers several advantages. It could eliminate the need for phantom calibration to measure bone density; it could improve the consistency of measurements, even with changes in soft tissue composition, which would allow tracking of bone formation over time; it could reduce radiation doses. All of this could facilitate and improve opportunistic osteoporosis screening, while streamlining clinical trials that rely on CT images for bone quantification. Therefore, the aim of this study was to quantitatively compare the effects of adipose tissue (fat), non-fat tissue (water) and air on phantom-dependent (QCT) and phantom-independent (DECT and PCT)-based BV/TV measurements and to evaluate the influence of reduced CTDI_v_ on DECT- and PCT-based measurements in trabecular bone samples.

## Materials and Methods

### Sample Preparation

Bovine trabecular bone was chosen to assess mean BV/TV with the help of three different CT scanners (SOMATON Definition Flash, NAEOTOM Alpha, µCT100) and four scanning methods (µCT as reference standard, QCT, DECT and PCT). Sixteen cylindrical trabecular bone biopsies (13 mm in diameter) of five bovine tibias were extracted from the sub-tibial plateau along the main tibial axis, using a hollow diamond-coated drill bit [25]. The extracted cylinders were cut to lengths ranging between 7.8 and 11 mm and covering a BV/TV range between 17 and 40%, based on µCT measurements. All samples were cleaned from bone marrow with an in-house cleaning protocol. Shortly, the samples were put for three minutes into an ultrasonic bath filled with a soapy solution and cleaned afterwards with a water jet and air pressure. These steps were repeated to remove as much of the marrow as possible. Finally, the samples were covered with a solution of 33% acetone and 67% water for 24 h to remove as much as possible of the remaining bone marrow. Visual inspection during the cleaning process and subsequent review of the µCT images were used to verify the removal of bone marrow.

### CT scanning and image reconstruction

**Fig. 1:**
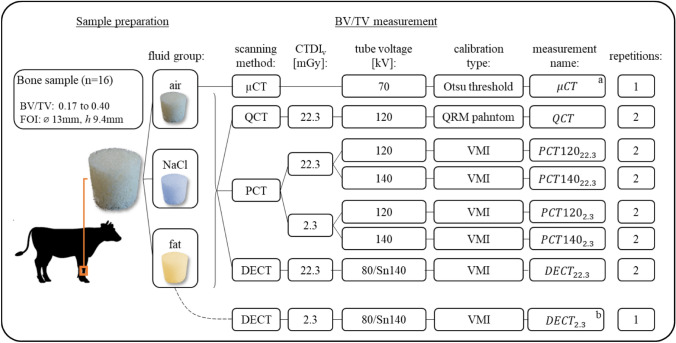
Study overview, showing the different bone volume-fraction (BV/TV) measurements using micro computed tomography (µCT) quantitative CT (QCT), photon counting CT (PCT) and dual energy CT (DECT). CTDI_v_ (in mGy) is the standardized measure for radiation dose output. The reconstructed images were calibrated for bone density measurements by segmentation using Otsu thresholding, a QRM phantom or phantom-less virtual monoenergetic image (VMI) reconstruction. a: µCT was used as reference BV/TV measurement. b: One additional measurement with fat was performed with DECT for a “low” CTDI_v_.

A total of seven distinct CT protocols for BV/TV measurement were defined (Fig. 1, Table 1). For the measurements: QCT_22.3_, PCT120_22.3_, PCT120_2.3_, PCT140_22.3_, PCT140_2.3_ and DECT_22.3_, the intertrabecular space of each sample was filled with three different soft tissue substitutes to replace the removed bone marrow one after the other. For that purpose, three different fluids were chosen: air (no filling), saline solution (0.9% NaCl), and a liquid adipose tissue surrogate mimicking the attenuation properties of fat as reported in the ICRU report 46 [26]. This resulted in three pairwise comparable fluid groups: (1) The air group (air), (2) the saline group (NaCl), and the adipose group (fat), with the purpose to evaluate the effect of the soft tissue composition on the BV/TV measurement. A separate measurement was conducted with DECT (DECT_2.3_, see Fig. 1) to assess the effect of dosage on DECT-based BV/TV measurement. This inquiry emerged during the study, after most DECT measurement scans were already performed.

**Table 1:** Overview of the CT scanner settings (tube voltage, beam filter, tube current, CTDI_v_ and slice thickness) for the different BV/TV measurement

Measurement name	QCT	DECT_22.3_		DECT_2.3_		PCT120_22.3_	PCT120_2.3_	PCT140_22.3_	PCT140_2.3_
Tube voltage [kV]	120	80/Sn140		80/Sn140		120	120	140	140
Tube current [mAs]	331	356		37		197	20	136	14
CTDI_v_ [mGy]	22.33	22.27		2.31		22.20	2.25	22.33	2.29
Slice thickness [mm]	0.6	0.6		0.6		0.4	0.4	0.4	0.4

A tin (Sn) beam filter was used for the 140 kV tube during DECT scanning. All images were reconstructed with a 512 × 512 matrix and a pixel spacing of 0.43 mm × 0.43 mm.

**Fig. 2:**
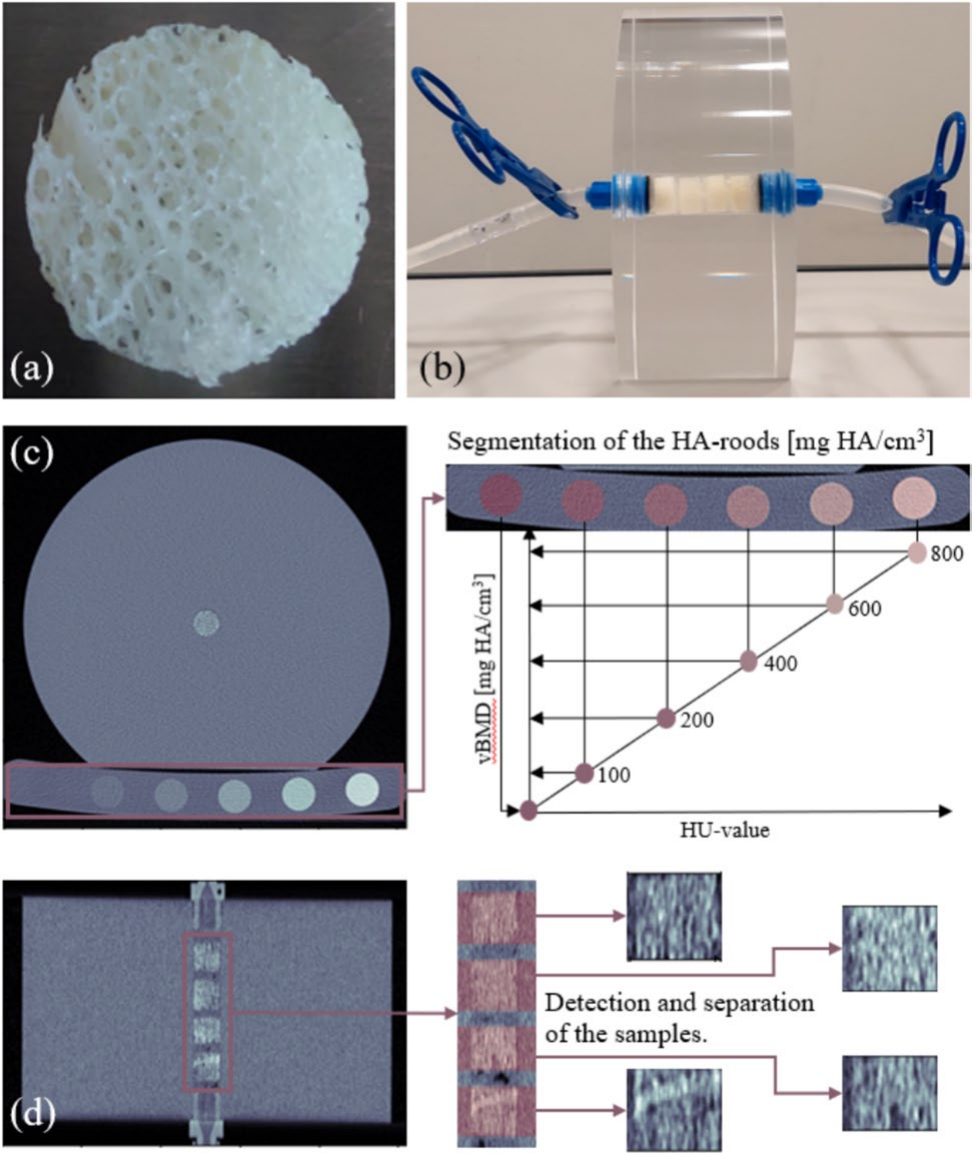
**a** Photograph of a cleaned trabecular bone specimen. Most of the soft tissue between the intertrabecular space could be removed with the described cleaning protocol. **b** Picture of the assembled sample holder for QCT, DECT, and PCT scanning. The cavity is assembled with four specimens (forming one stack of samples) and filled with fluid. After venting over a system of silicon tubes and two syringes, the system was closed airtight. **c** Schematic visualization of the segmented phantom rods, followed by the construction of the HU to vBMD mapping function. For QCT, DECT and PCCT scanning, the QRM calibration phantom was placed between sample holder and CT table. **d** Visualization of the image processing steps to allocate and separate the four bone samples, which were assembled in the PMMA sample holder for QCT, DECT and PCT image acquisition.

To ensure compliance between each scanning method, QCT, DECT, and PCT scans were performed with the same experimental setup including an in-house customized disk shaped PMMA sample holder (see Appendix, Fig. 7) and a standard QCT bone density calibration phantom (BDC/6-200, QRM GmbH, Moehrendorf, Germany). This phantom is equipped with six phantom rods, each containing a different concentration of hydroxyapatite: 0, 100, 200, 400, 600, and 800 mg HA/cm^3^. The samples were assembled in groups of four (one sample stack) inside the cavity of the PMMA sample holder (Fig. 2). Afterward the cavity was closed, filed with the appropriate fluid, and vented to ensure that the intertrabecular space of the samples was filled with the fluid. The calibration phantom was positioned between the sample holder and the CT table, enabling QCT calibration [3]. This setup also aimed to mimic additional soft tissue and bone, thereby simulating beam hardening effects commonly observed in clinical scans. QCT and DECT scans were conducted, without moving the setup, on the same clinical dual-source, 128-slice multi-detector row CT scanner (SOMATON Definition Flash, Siemens Healthineers, Forchheim, Germany). PCT scans were performed on a clinical dual-source with a new photon-counting detector system (NAEOTOM Alpha, Siemens Healthineers, Forchheim, Germany). After scanning with a specific fluid, the samples were cleaned and reassembled with the next fluid.

As reference, high-resolution µCT scans with air were acquired, for each sample separately, on a µCT100 (Scanco Medical, Switzerland), operating at a spatial resolution of 20 µm, with tube voltage and current settings at 70 kV and 85 µA, respectively [27]. For this purpose, the scanner’s standard sample holders were used.

### Bone Volume-Fraction Assessment

BV/TV is given in percentage as the ratio between the amount BV and its TV, inside a volume of interest (VOI). To determine the BV/TV resulting for each sample form the measurement, the samples were first identified within the reconstructed images through a dedicated, in-house developed segmentation pipeline (see Fig. 2b), implemented in Python (version 3.8). In all images of the sample stacks, the position of a VOI of fixed diameter and sample-specific height was matched to that of each sample in the image, denoted as VOI_*i*_, where *i* ∈ {1, 2, ..., 16}. Each VOI_*i*_ was characterized by a unique height (*H*_*i*_) and a constant diameter (*D*) of 12.5 mm, that was slightly smaller than the actual volume of a sample. This consistent dimensioning of VOI_*i*_, as specified by *H*_*i*_ and *D*, was applied across all BV/TV measurements to ensure uniformity in volume for BV/TV assessment. With this approach, the different image resolutions could be considered, thus minimizing partial volume effects.

#### µCT Using Otsu Thresholding

For µCT no calibration phantom was used. The BV/TV was determined by segmenting the voxels inside the VOI_*i*_ into bone and non-bone voxels by defining a global Otsu threshold according to their CT value, utilizing the Python library skimage. To ensure consistency across all samples, the threshold was established by calculating an average Otsu threshold across all 16 samples, allowing for the application of the same threshold for all samples. This segmentation technique differentiates the BV from other materials present in the intertrabecular space. Consequently, BV/TV was calculated by dividing the total number of segmented bone voxels (BV) by the total number of voxels within the VOI_*i*_ (TV).

#### QCT with Calibration

For each sample, a distinct HU to vBMD conversion function was established using the calibration phantom to adjust for variability between scans. To address this, the six phantom rods were segmented within each axial CT-slice along the longitudinal axis of the sample. The HU values attributed to each rod were then averaged across the length of the sample’s VOI_*i*_, denoted as *H*_*i*_. Subsequently, a mapping function specific to each sample and acquisition was developed through a linear regression analysis between the HU values and the volumetric vBMD [mgHA/cm^3^] associated with the phantom rods.

The mean vBMD in the VOI was approximated using the HU value averaged over all voxels in the VOI and the specific mapping function. The vBMD-based BV/TV was subsequently determined by dividing the mapped vBMD_*i*_ by 1200 mg/cm^3^ [7, 12].

#### PCT and DECT Using Mono Chromatic Image Reconstruction

HU values exhibit a strong dependency on the photon energy level *E*. Utilizing VMIs enables the direct computation of BV/TV through a designated system of equations given by the materials composition and the photon energies.

1 $$\text{HU}\left(E\right)=\sum_{i=\text{component}}{\rho }_{i}{\alpha (E)}_{i}{\upphi }_{i}-\Theta$$

${\phi }_{i}$ is the volume fraction, ${\rho }_{i}$ is the density, ${\alpha (E)}_{i}$ the energy-dependent X-ray mass attenuation coefficient of component *i* normalized by the mass attenuation coefficient of water and $\Theta$ a constant approximately equal to 1000 for most CT scanners [11]. Nickoloff et al. [11] adapted this equation for trabecular bone by leveraging its composition of collagen, calcium hydroxyapatite, water, red marrow, and adipose tissue. Further simplification is done by consolidating water and red bone marrow into a category termed non-adipose tissue (represented here as NaCl), justified by the similar density and photon-attenuation properties of these two components. Furthermore, collagen and HA were merged into a composite termed the bone matrix material, characterized by an average density of 1.92 g/cm^3^. This integration resulted in the formulation of the following general equation applicable to our sample groups.

2 $$\begin{aligned} {\text{HU}}\left( E \right) & = \mu \left( E \right) \cdot \frac{{{\text{BV}}}}{{{\text{TV}}}} + \alpha \left( E \right)_{{{\text{fat}}}} \cdot \rho_{{{\text{fat}}}} \cdot \phi_{{{\text{fat}}}} \\ & \quad + \alpha \left( E \right)_{{{\text{NaCl}}}} \cdot \rho_{{{\text{NaCl}}}} \cdot \phi_{{{\text{NaCl}}}} + \alpha \left( E \right)_{{{\text{air}}}} \cdot \rho_{{{\text{air}}}} \cdot \phi_{{{\text{air}}}} - {\Theta } \\ \end{aligned}$$

In our study, it was assumed that 0.9% saline solution (NaCl group) has a similar attenuation characteristic as the non-adipose tissue. $\mu \left(E\right)$ is the energy-dependent normalized mass attenuation coefficient of the composite collagen and HA in relation to its density, using the assumption given by Nickoloff et al. [11]. The energy-dependent CT coefficients ${\alpha (E)}_{i}$ were derived from the components linear attenuation coefficients reported in National Inst. of Standards and Technology [28]. The density of 0.9% saline solution was calculated to be approximately 1.005 g/cm^3^, density of air to 0.0013 g/cm^3^, of collagen was set to 1.2 g/cm^3^ [29], of fat to 0.92 g/cm^3^, of HA to 3.06 g/cm^3^ and of the matrix material to 1.92 g/cm^3^ [11].

For each of the three fluid groups (air, fat and NaCl), the equation can be simplified because, besides BV/TV, only one additional material is present, while the quantities of the others are assumed to be negligible. In the equations for NaCl and fat, the air component was not considered negligible due to the expectation that small air bubbles might remain after filling the sample holders. Leading to the following considerations for each fluid group:

3 $$\text{NaCl group}: {\phi }_{\text{fat}}=0$$

4 $$\text{FAT group}: {\phi }_{\text{NaCl}}=0$$

5 $$\text{AIR group}: {\phi }_{\text{fat}}=0\text{ and }{\phi }_{\text{NaCl}}=0$$

The DECT- and PCT-based VMI, were reconstructed using the software, syngo.via version 8.7 (Siemens Healthineers, Forchheim, Germany). VMIs were produced across a spectrum ranging from 40 to 140 keV. Iterations with different combinations showed most stable results for the combination of the four energy levels: 40, 60, 80, and 100 keV.

Using conservation of volume of the materials inside the VOI and the four VMI gives an overdetermined system of equations with 5 equations and three unknowns for each fluid group: BV/TV, ${\phi }_{\text{fluid}}$ and ${\phi }_{\text{AIR}}$, which was solved with a weighted linear least-square algorithm with bounds [0, 1] on the variables (SciPy scipy.optimize.lsq_linear).

### Statistical Evaluation

All statistical analysis were performed with R. *P*-values < 0.05, 0.01, 1e−3 or 1e−4 are indicated with *, **, *** or ****, respectively.

#### Accuracy

The accuracy of a measurement was obtained by its mean error averaged over all 16 samples and 2 repetitions. To do so, the individual delta ${\Delta }_{i}^{n}$ to the reference (given by the µCT measurement) of each sample *i* = {1 … 16} and repetition *n* = {rep1, rep2} was calculated by

6 $$\Delta_{i}^{n} = {\text{BV}}/{\text{TV}} _{{{\text{scanning}}\,{\text{method}},\,i}}^{n} - {\text{BV}}/{\text{TV}}_{{\upmu {\text{CT}}}},$$

averaged over the number of repetitions gives the mean error $\overline{{\varepsilon }_{i}}$ for sample *i*

7 $$\overline{{\varepsilon }_{i}} = \frac{\left({\Delta }_{i}^{\text{rep}1}+{\Delta }_{i}^{\text{rep}2}\right) }{2}$$

and finally, $\overline{{\varepsilon }_{i}}$ averaged over the number of samples gives the mean error. A one sample *t*-test with a hypothesized mean error equals to zero was utilized to test if there is a significance difference between measured BV/TV and the reference (BV/TV_µCT_), while considering the variability of the data. This two values, mean error and *t*-test were used to assess the effect of the different soft tissue substitutes on the BV/TV measurements.

#### Reproducibility

Reproducibility was assessed using the method outlined by Glüer et al. [30]: the absolute average delta $\left|\overline{{\Delta }_{i}}\right|$ over the two repetitions was calculated for each sample i:

8 $$\left|\overline{{\Delta }_{i}}\right| = \frac{\left(\left|{\Delta }_{i}^{\text{rep}1}\right|+\left|{\Delta }_{i}^{\text{rep}2}\right|\right) }{2}$$

Short term precision, given by the standard deviation (SD_*i*_) of each sample *i,* was calculated. Finally, the precision error of each measurement ($\overline{\text{SD} }$) is given by the root mean square error, which is given by the arithmetic mean of the in individual sample’s variances ${\text{SD}}_{i}^{2}$.

#### ANOVA and Pairwise Comparison

ANOVA and post hoc tests were employed to assess the impact of various factors: scanning method (QCT, DECT, PCT), fluid group (NaCl, fat, air), CTDI_v_ (for DECT and PCT: 2.3, 22.3), and tube voltage (for PCT: 120, 140), on the BV/TV measurements mean error. These factors served as independent variables and the mean error as the dependent variable. This evaluation differentiated between two scenarios: the *clinical* and the *post-mortem* scenario. The *clinical* scenario was designed to mimic the conditions of a clinical setting, focusing on validating BV/TV measurements where air inclusions are absent.

In the initial phase of the *clinical* scenario, the aim was to determine whether there was a discernible difference between NaCl and fat across each scanning method, maintaining a consistent CTDI_v_ (22.3) and comparable tube voltage. A two-way ANOVA was conducted for this purpose, with fluid (NaCl and fat) and BV/TV measurements (PCT120_22.3_, DECT_22.3_, QCT_22.3_) serving as independent variables.

In the subsequent step, the investigation focused on the effects of reducing the CTDI_v_ and adjusting the tube voltage on PCT-based measurements. To evaluate these factors, a three-way ANOVA was executed, with fluid (NaCl and fat), voltage (120 and 140), and CTDI_v_ (22.3 and 2.3) as the independent variables.

In addition, a one-way ANOVA was implemented to evaluate the impact of CTDI_v_ on DECT-based measurements, limited by the fact that the low-CTDI_v_ measurement was solely utilized for the fat group. This examination designated CTDI_v_ (22.3 and 2.3) as the sole independent variable.

The *post-mortem* scenario is reflective of conditions found in a dead body, where the presence of air inclusions is not neglectable. In this context, the concern for dose-related harm is mitigated, as additional radiation exposure poses no risk to deceased specimens. Accordingly, a two-way ANOVA was executed to investigate the interplay between fluid (NaCl, fat, and air) and BV/TV measurement (DECT_22.3_, PCT120_22.3_, and PCT140_22.3_), which were defined as the independent variables for this analysis.

The results were reported by the *p*-value and the F-test: F(*x*, *y*), with *x* and *y* denoting the degrees of freedom in the numerator and denominator, respectively. Significant main effect were followed up by pairwise comparisons between different level of groups by applying a Bonferroni adjustment and using the function emmean_test(). The following tests were carried out to check whether the data met the assumption considered for the one-way, two-way and three-way ANOVAs: Potential extreme outliers in each group were assessed using box plot methods implemented in the R function identify-outliers() (rstatix package); normality was assessed by analyzing the model residuals using QQ plot and Shapiro-Wilk’s normality test (shapiro_test()); homogeneity of variances was assessed by Levene’s test (levene_test()).

## Results

For all ANOVA tests conducted, the data met the prerequisite assumptions of normality and homogeneity of variances.

### Accuracy and Repeatability

Good reproducibility was found with absolute precision error of 0.7% or lower for all BV/TV measurements (Table 2). An exception was observed with QCT air, which displayed a notably higher absolute precision error of 2.66% BV/TV. Despite this increase, the measurements for QCT air were still seen as reproducible.

**Table 2:** Overview of the results, grouped by the eight BV/TV measurements and three fluid groups (fat, air and NaCl)

BV/TV measurement name	Fluid group	$\frac{1}{N}\sum_{i=1}^{N=16}{\Delta }_{i}^{\text{rep}1} [\%]$	$\frac{1}{N}\sum_{i=1}^{N=16}{\Delta }_{i}^{\text{rep}2 }[\%]$	Mean error accuracy [%]	One sample *t*-test *p*-value	$\overline{\text{SD} }$ precision [%]
DECT_22.3_	Fat	0.02	0.23	0.13	0.577	0.44
DECT_22.3_	Air	0.91	0.87	0.89	0.003	0.61
DECT_22.3_	NaCl	− 0.44	− 0.49	− 0.46	0.111	0.53
DECT_2.3_	Fat	N/A	1.33	1.33	1.336E−05	N/A
PCT120_22.3_	Fat	0.59	0.10	0.34	0.186	0.46
PCT120_22.3_	Air	− 0.53	− 0.18	− 0.35	0.060	0.42
PCT120_22.3_	NaCl	0.69	− 0.27	0.21	0.493	0.64
PCT120_2.3_	Fat	0.27	− 0.14	0.07	0.787	0.44
PCT120_2.3_	Air	− 1.34	− 0.84	− 1.09	4.392E−05	0.39
PCT120_2.3_	NaCl	0.71	0.01	0.36	0.263	0.70
PCT140_22.3_	Fat	− 0.14	− 0.14	− 0.14	0.545	0.24
PCT140_22.3_	Air	− 0.78	− 0.31	− 0.54	0.010	0.42
PCT140_22.3_	NaCl	− 0.32	− 0.67	− 0.49	0.102	0.50
PCT140_2.3_	Fat	− 0.50	− 0.15	− 0.33	0.163	0.36
PCT140_2.3_	Air	− 1.63	− 1.01	− 1.32	1.891E−05	0.52
PCT140_2.3_	NaCl	− 0.26	− 0.69	− 0.48	0.102	0.45
QCT	Fat	− 1.02	− 0.84	− 0.93	0.008	0.46
QCT	Air	− 41.48	− 40.44	− 40.96	1.733E−15	2.66
QCT	NaCl	0.74	0.94	0.84	0.018	0.67

The table showcases the average delta to the reference BV/TV [%] for each repetition (${\Delta }^{\text{rep}1}$ and ${\Delta }^{\text{rep}2}$) over all 16 samples, the mean error (accuracy) of the measured BV/TV [%], the *p*-value of the *t*-test and the precision error of the measured BV/TV [%] for each measurement ($\overline{\text{SD} }$). For DECT_2.3_ the measurement was done only once so the precision could not be calculated

The *t*-test revealed that the measurement mean errors were highly significant (*p*-value < 0.05, Table 2) and differed substantially from zero for DECT_22.3_ air, DECT_2.3_ fat, PCT120_2,3_ air, PCT140_22.3_ and PCT140_2.3_ air, and QCT for all three fluids. For all remaining measurements, the differences were non-significant.

Upon excluding the air group, PCT140_22.3_, and PCT140_2.3_, demonstrated the lowest mean error (Table 2), averaging 0.37% and 0.41% BV/TV, respectively, relative to other measurements. Conversely, QCT_22.3_ and PCT120_2.3_ exhibited the highest precision errors, both with a mean value of 0.57% BV/TV.

### QCT, DECT and PCT120 at CTDI_v_ = 22.3; Clinical Scenario

- BV/TV measurement: DECT_22.3_, PCT120_22.3_, QCT
- Fluid: fat, NaCl.

There were no significant main effects of the BV/TV measurement or the fluid on the mean error. However, a significant interaction effect between the fluid and BV/TV measurement was observed, F(2,90) = 9.97, *p*-value < 0.001.

**Fig. 3:**
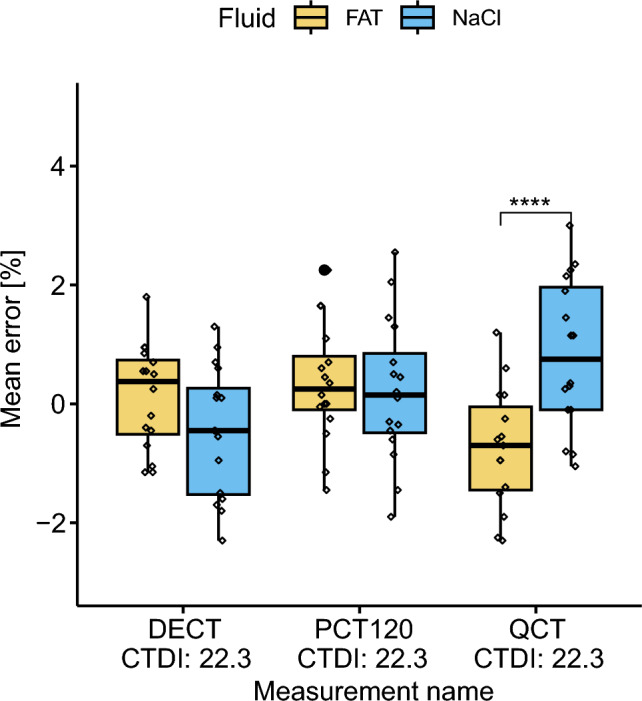
Boxplot of mean error grouped by the BV/TV measurement and fluid. Only the QCT-based BV/TV measurement significantly differed for the different fluids. No differences could be found for DECT_22.2_ and PCT_22.3_.

Pairwise comparison yielded a significant difference for QCT in the mean error between the fat and NaCl, with a *p*-value = 2.28e−05 (Fig. 3). Specifically, the absolute mean error for QCT fat was larger than that for QCT NaCl. When assessing the impact of the BV/TV measurement on the mean error with each fluid, the mean error for QCT was significantly different compared to DECT_22.3_ and PCT_22.3_ across the fat group. And significantly different to DECT_22.3_ across the NaCl group (Fig. 3).

Between DECT_22.3_ and PCT_22.3_, no significant difference was found, and the type of fluid did not affect the mean error significantly.

### PCT120 and PCT140; the “low” CDTI_v_; Clinical Scenario

- Scanning method: PCT
- CTDI_v_: 22.3, 2.3
- Tube voltage: 120, 140
- Fluid: fat, NaCl.

**Fig. 4:**
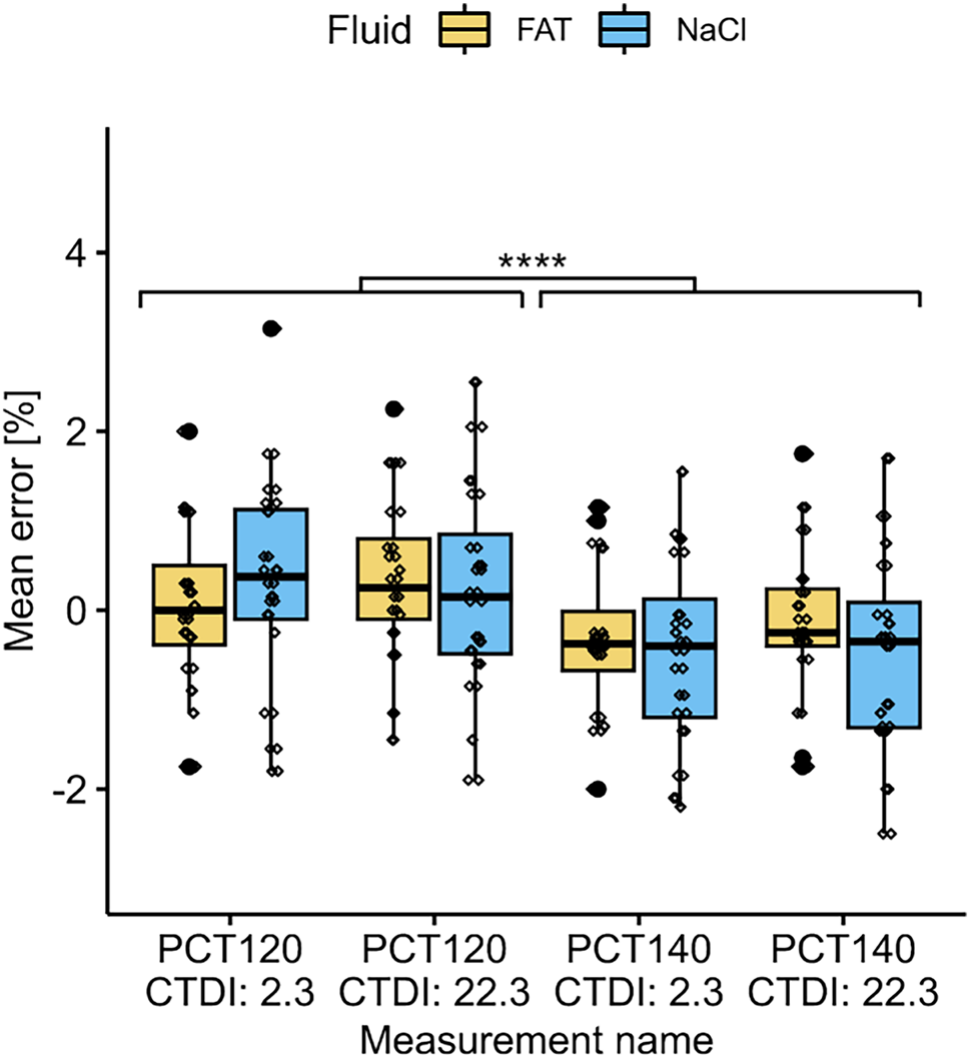
Boxplot of mean error grouped by the BV/TV measurement and fluid. Between same voltage groups, no difference between 22.3 and 2.3 CDTI_v_ could be detected. In contrast, a significant difference between the two voltage groups (120, 140) was recognized.

The validation showed significant main effects for tube voltage on the measurement error, F(1,248) = 21.448, *p*-value = 8.00e−02. No further main effects or interaction could be found (Fig. 4). Pairwise comparison showed that the mean errors are significantly different between 140 and 120 tube voltage, *p*-value = 5.248e−06, independent form CTDI_v_. No significant differences between 22.3 and 2.3 CTDI_v_ within a voltage group was found.

### DECT; the “low” CDTI_v_; Clinical Scenario

- Scanning method: DECT
- CTDI_v_: 22.3, 2.3
- Fluid: fat.

**Fig. 5:**
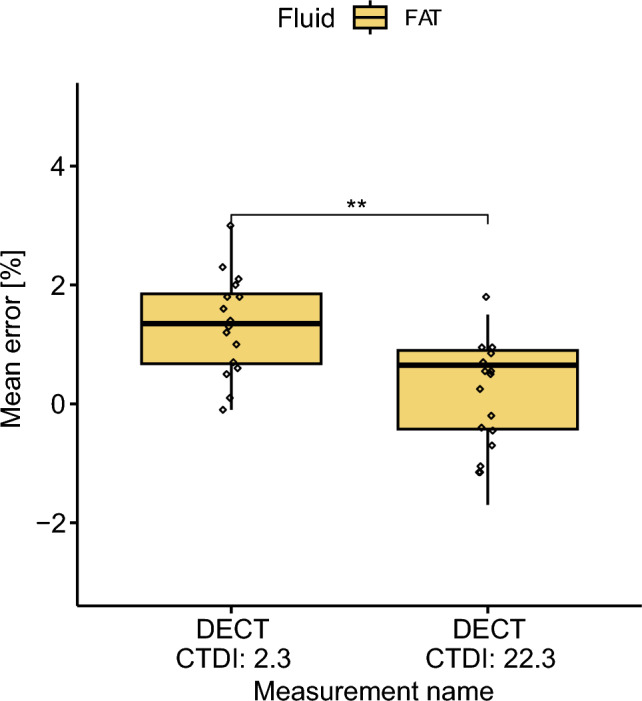
Boxplot of mean error grouped by the BV/TV measurement. DECT showed a significant difference between 2.3 and 22.3 CTDI_v_. DECT_2.3_ led to higher mean error than DECT_22.3_.

The validation showed significant main effects of the CTDI_v_ on the mean error, F(1, 30) = 11.29, *p*-value = 0.002 (Fig. 5). A lower CTDI_v_ leads to a significant increase of the mean error. Indeed, the mean error for DECT_2.3_ increases compared to DECT_22.3_.

### The Presence of Air; Post-Mortem Scenario

- BV/TV measurement: DECT_22.3_, PCT120_22.3_, PCT140_22.3_
- Fluid: fat, NaCl, air.

The analysis revealed that the choice of BV/TV measurement had a statistically significant influence on the mean error, F(2, 135) = 4.67, *p*-value = 0.01. Moreover, a notable interaction effect between the fluid and the BV/TV measurement on mean error was detected, F(4, 135) = 4.55, *p*-value = 0.002.

**Fig. 6:**
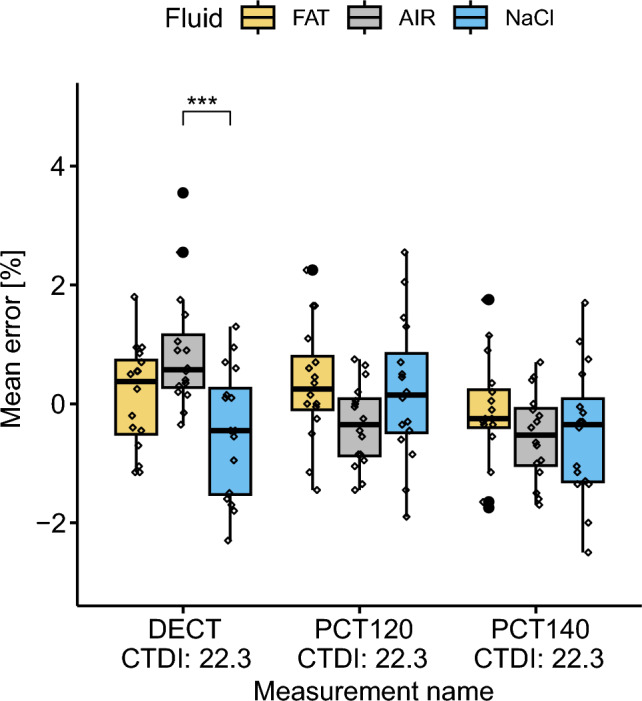
Boxplot of mean error grouped by BV/TV measurement and subgrouped by fluid. A significant difference in mean error was recognized for DECT_22.3_ between air and NaCl.

**Fig. 7:**
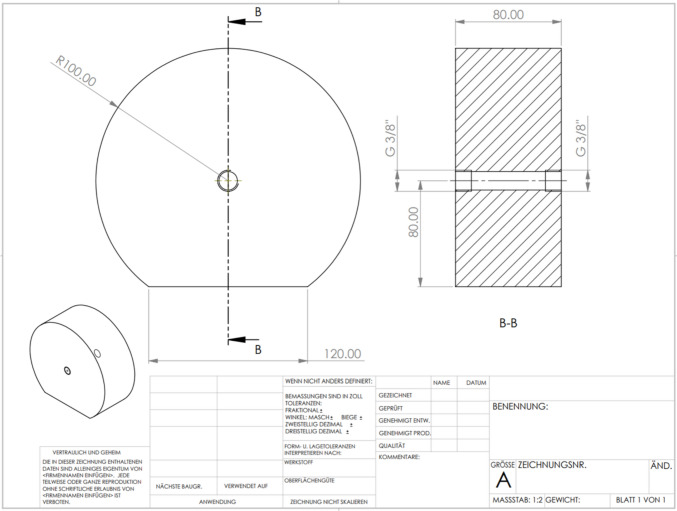
Drawing of the PMMA sample holder for QCT, DECT and PCT scanning.

Simple main effects analysis pinpointed that within DECT_22.3_, the fluid significantly affected the mean error, (F(2, 135) = 7.67, *p*-value = 6.97e−4). Pairwise comparison refined these insights by identifying that the mean error associated for air using DECT_22.3_ (Fig. 6) was significantly different compared to NaCl (*p*-value = 4.42e−4). This highlights the sensitivity of DECT_22.3_ to the fluid type used, especially if air is involved. Contrarily, for PCT120_22.3_ and PCT140_22.3_, the fluid did not significantly alter the mean error, underscoring the robustness of PCT.

Additionally, significant differences in mean error were detected between DECT_22.3_ and PCT140_22.3_ (*p*-value = 0.021), which might suggest differing capabilities of these methods to assessing BV/TV.

## Discussion

This study aimed to compare the accuracy of BV/TV measurements using QCT against phantom-less DECT- and PCT-based measurements on sixteen cylindrical trabecular bone specimens from bovine tibias. The samples were modified by replacing the bone marrow with three different media to simulate different compositions of the bone marrow and to evaluate its effects on seven different BV/TV measurements using µCT images as a reference standard. This experimental setting enabled a comprehensive statistical analysis to determine the reliability and reproducibility of each BV/TV measurement. The study differentiates between two use cases: the *clinical/in vivo* and *post-mortem/ex vivo* scenario.

The reproducibility of the applied experimental setup was confirmed, demonstrating its reliability and usability for the comparison of different techniques for BV/TV measurement and the evaluation of the impact of the filling material in the intertrabecular space on the measurement.

For the *clinical* scenario with CTDI_v_ = 22.3, the mean error of QCT within either NaCl or fat was found to be approximately ± 1% BV/TV (Table 2, mean error), which is comparable with the error range of a DEXA measurement (1–2%), probably clinically not relevant, and indicates a relatively high level of accuracy for both fluid groups. However, despite this, QCT measurements exhibited a significant deviation from the reference measurement for both NaCl and fat (Table 2, one-sample *t*-test). Specifically, within the fat group, the BV/TV was underestimated, whereas in the NaCl group, it was overestimated. Furthermore, this discrepancy was highlighted by a clear interaction between the fluid type and QCT in the pairwise comparison analysis and a significant difference between NaCl and fat (Fig. 3). In comparison, for DECT and PCT, the mean error was in the range of ± 0.3% and ± 0.1% BV/TV, respectively. There were no significant differences in the BV/TV measurement between the fat and NaCl (Table 2, one-sample *t*-test), indicating that these measurements are more robust against variations in fluid composition at this CTDI_v_. They also do not require a calibration phantom, which remains a major drawback of QCT.

So far, technical limitations and “high” radiation dose of CT scanners prevented their clinical application in evaluating bone density. Nevertheless, especially photon-counting detectors used for PCT scans have improved and stabilized spectral measurements and enables the reduction of the radiation dose without losing information [14, 21, 31, 32].

This was also recognized in our results. In contrast to DECT (± 0.6% BV/TV, between 22.3 and 2.3 CTDI_v_), lowering the CTDI_v_ did not impact the accuracy of the PCT measurements (± 0.1% BV/TV, between 22.3 and 2.3 CTDI_v_). There was a significant difference in the BV/TV measurement when changing the tube voltage from 120 to 140. Nevertheless, the mean errors to the reference measurement are in the range of ± 1% (Table 2), which is again probably not relevant in a clinical setting. Those finding underscores the robustness, versatility, and potential of PCT for clinical applications like for osteoporosis screening or bone density assessment for surgical planning. It offers the ability to reduce radiation exposure without compromising measurement reliability. Therefore, PCT appears as a potential technique for opportunistic BV/TV screening, even when various scanning protocols are being employed. Our results show, that these technologies, especially measurements originated from “low” CTDI_v_ PCT reconstructions, offer an alternative to current clinical standards, such as dual X-ray absorptiometry (DXA), QCT and high-resolution peripheral quantitative CT (HR-pQCT) [3, 7].

For the *post-mortem* scenario, where air could cause issues, the QCT-based BV/TV measurement was less robust against exchange of the filling material in the intertrabecular space than DECT and PCT. For air, the mean error of QCT is considerable high (over ± 20% BV/TV), and precision gets worse (Table 2), which underscores its substantial inaccuracy under such conditions. Therefore, QCT appears less suitable as a technique whenever air or a fluid with similar X-ray attenuation characteristics is present, accordingly the presence of large air bubbles must be avoided for *ex vivo* quantitative measurements. DECT- (± 0.7% BV/TV) and PCT (± 0.3% BV/TV)-based BV/TV measurements were more robust. Nevertheless, an influence of the fluid type on the DECT measurement was observed, with the air showing significant differences to NaCl and fat. However, no interaction was found between PCT-based measurements and fluid (Fig. 6). Although DECT, PCT120 and PCT140 measurements with air significantly diverged from the reference standard, their mean errors were relatively low, at 0.89%, − 0.35%, − 0.54% BV/TV, respectively. This maintains their status as suitable methods for post-mortem BV/TV estimations.

Coming back to our hypothesis. First, it has been demonstrated that the presence of fat systematically resulted in lower BV/TV values when measured using QCT. Contrarily, the estimation of BV/TV based on VMIs reconstructed from DECT or PCT remained unaffected by the presence of fat within the intertrabecular space, confirming hypothesis (1). Furthermore, QCT NaCl showed increased BV/TV compared to the reference measurement, which could be due to the higher X-ray attenuation of the 0.9% NaCl solution compared to CT water (CT water is a component of the calibration phantom for setting the HA concentration in the phantom rods and is equivalent to H_2_0). This shows the strong dependence of the QCT measurement accuracy on the material composition of the calibration phantom used. Second, PCT measurements were not influenced by CTDI_v_, thus confirming hypothesis (2). Lastly, hypothesis (3) is partially fulfilled, since PCT and DECT were impacted in the presence of air. In general, DECT exhibited less robustness compared to PCT, primarily due to the scintillator detectors’ lower sensitivity to low-energy photons in DECT systems as opposed to the new photon-counting detectors used in PCT [22].

The findings of the performed experiment highlight the significant influence of fluid composition on the accuracy of QCT-based BV/TV measurement. This observation aligns with the results from comparable studies [14, 17, 33], which also emphasize the sensitivity of QCT to variations in fluid composition surrounding the bone. In contrast, VMI-based BV/TV measurements, such as those obtained through DECT and PCT, demonstrate robustness against changes in soft tissue composition. These BV/TV measurements provide a valuable addition to traditional bone density evaluation methods like DEXA. Particularly, PCT stands out due to its dose-effective scanning capabilities and straightforward access to spectral data.

Several limitations need to be considered. First, bovine bone was used instead of human samples due to availability reasons. The samples size (*n* = 16) was relatively small but a good compromise to save preparation time between the different fluid groups and scanning methods. Furthermore, for the VMI-based BV/TV measurement, the composition of the bone ECM was assumed to be constant [11], which is comparable to the assumption of constant tissue mineralization for the QCT-based BV/TV calculation. BV/TV derived from segmented µCT images is threshold dependent. However, a uniform threshold was used for all samples and the resolution of 20 µm enables to resolve the trabecular tissue, which makes it as a reasonable technique to use as a reference standard measurement. It cannot be completely excluded that small amounts of bone marrow were not removed during the cleaning process. Minor residues in the pores are unlikely to have a significant impact on the conclusions of the study, as they have a small volume relative to the bone matrix and pores. The samples were embedded in a PMMA sample holder, which was positioned on top of a calibration phantom equipped with HA rods during the CT scanning process. This setting only partially mimicked a human body and featured limited beam hardening effects which may occur in a clinical scenario. It does not consider differences in the thickness of the soft tissue surrounding the bone. This can introduce beam hardening effect and potentially influencing the clinical accuracy of the BV/TV measurement. However, this was not the subject of the study. The experimental setup was designed to facilitate the precise comparison of different BV/TV measurements within a controlled environment and not to validate the effect of the thickness of the surrounding soft tissue.

All scans were conducted on scanners from the same manufacturer, and VMI reconstruction was only performed with one software. While this removes some sources of variation, the effect of the different manufactures and software on the performance of VMI-based BV/TV measurements needs to be further investigated.

Nevertheless, this experimental setup enables a pairwise statistical comparison under controlled and reproducible conditions, yielding results that align with those found in similar studies. The consistency of these results with prior phantom studies not only validate the employed experimental methodology but also contributes valuable insights into the strengths of the different scanning methods for bone density measurements. The used setup can be further used to include and validate other image variables as well as CT scanners of different brands to the comparison.

In summary, this study provides a reproducible experimental setup to quantitatively investigate the influence of soft tissue composition in the intertrabecular space on the sensitivity, accuracy, and robustness of different scanning methods for BV/TV measurements and emphasizes the complexity of such in the field of medical imaging.

Despite these complexities, VMIs, reconstructed form DECT and PCT were not sensitive to soft tissue alteration and thereby enabling a more precise BV/TV measurement, while QCT led to clear differences between fat and NaCl. Furthermore, *post-mortem* PCT may provide accurate BV/TV estimation in the presence of air. At last, VMIs-based BV/TV measurements, especially based on reconstructions form “low” radiation dose PCT scans, stand out as a robust alternative to DEXA or hr-pQCT.

Overall, the new generation of spectral CT scanners offer a pathway to more accurate and reliable BV/TV assessments, also in opportunistic settings. This advancement holds significant promise for improving the diagnosis and monitoring the condition of bone disease, thereby enhancing patient care through more informed clinical decision-making, as well as for the field of forensic and anthropology.

## Appendix

See Fig. 7 and Tables

**Table 3:** Overview of the Bone Volume to Total Volume (BV/TV [%]) ratios for each sample, assessed across the seven CT protocols used for BV/TV measurement during the initial round of measurements (rep1).

	Sample	uCT reference	QCT_22.3_			DECT_22.3_			DECT_2.3_	PCT_22.3_			PCT_22.3_			PCT_2.3_			PCT_2.3_		
		Air (%)	Fat (%)	NaCl (%)	Air (%)	Fat (%)	NaCl (%)	Air (%)	Fat	Fat (%)	NaCl (%)	Air (%)	Fat (%)	NaCl (%)	Air (%)	Fat (%)	NaCl (%)	Air (%)	Fat (%)	NaCl (%)	Air (%)
First measurement cycle: rep1	T1_1	30.50	31.40	33.60	− 10.20	32.00	31.50	31.00	N/A	32.10	32.00	30.60	32.40	32.50	30.90	31.90	32.00	30.00	32.00	33.60	30.10
	T1_2	17.00	15.30	18.80	− 31.00	17.60	17.20	18.70	N/A	16.70	16.80	17.50	16.90	17.00	17.60	17.10	16.90	16.60	16.90	17.00	16.70
	T3_1	30.40	30.20	30.90	− 5.70	31.10	29.90	31.60	N/A	30.50	28.80	29.90	30.80	29.00	30.10	30.30	28.90	29.70	30.40	30.10	29.30
	T4_1	24.60	24.60	27.70	− 21.20	25.80	25.70	24.60	N/A	25.60	25.50	24.70	25.80	26.30	24.80	25.40	25.20	24.00	25.40	25.70	24.20
	T4_2	28.90	28.40	29.40	− 15.20	30.10	28.60	26.30	N/A	28.90	26.90	30.10	29.00	27.80	30.20	28.70	27.80	29.80	29.00	28.80	29.60
	T4_3	30.00	28.80	29.90	− 6.30	29.80	28.20	31.20	N/A	28.60	28.20	28.60	29.00	28.70	28.70	28.60	28.00	27.80	28.80	29.30	27.90
	T5_1	24.90	23.40	22.80	− 22.30	25.10	21.90	23.30	N/A	25.00	24.30	25.00	25.30	24.70	25.10	25.00	24.30	24.10	24.90	24.30	24.30
	T5_2	28.80	26.00	28.00	− 11.50	27.10	28.50	29.80	N/A	28.10	27.70	28.30	28.40	27.90	28.40	28.40	27.40	27.40	28.40	29.40	27.70
	T5_3	20.50	16.50	19.90	− 22.20	19.10	19.00	22.90	N/A	19.00	18.30	20.10	19.10	18.60	20.20	18.80	18.60	19.40	18.80	19.30	19.70
	T5_4	17.50	15.30	17.70	− 21.40	17.10	17.00	22.00	N/A	17.40	17.10	18.70	17.60	17.40	18.70	17.20	16.80	18.10	17.50	17.70	18.20
	T5_5	31.50	32.00	32.80	− 1.90	32.30	31.60	34.10	N/A	31.90	32.20	31.80	32.20	32.50	32.00	32.70	32.30	31.40	32.30	32.50	31.50
	T5_6	40.30	40.60	41.30	10.30	39.20	38.00	40.70	N/A	38.60	38.50	39.10	38.60	38.60	39.40	39.30	38.60	38.20	38.80	38.30	38.50
	T5_7	22.30	21.40	23.40	− 24.90	23.10	22.40	21.90	N/A	22.20	21.90	22.10	22.50	22.40	22.10	22.20	21.90	21.60	22.30	22.00	21.70
	T6_1	26.40	25.80	28.90	− 17.00	27.10	27.00	27.20	N/A	26.60	26.10	25.50	27.00	26.90	25.60	26.30	26.00	25.10	26.40	28.40	25.10
	T6_2	27.40	28.00	30.50	− 14.20	28.70	28.50	28.10	N/A	28.20	27.60	25.90	28.50	28.00	26.00	28.20	27.60	24.80	28.20	28.00	25.60
	T6_3	36.80	36.60	37.20	5.50	36.30	35.00	38.30	N/A	36.10	35.20	34.90	36.30	35.20	35.10	35.30	34.40	33.70	35.50	33.60	34.30

^3^ and

**Table 4:** Overview of the Bone Volume to Total Volume (BV/TV [%]) ratios for each sample, assessed across the seven CT protocols used for BV/TV measurement during the second round of measurements (rep2).

	Sample	uCT reference	QCT_22.3_			DECT_22.3_			DECT_2.3_	PCT140_22.3_			PCT120_22.3_			PCT140_2.3_			PCT120_2.3_		
		Air	Fat (%)	NaCl (%)	Air (%)	Fat (%)	NaCl (%)	Air (%)	Fat (%)	Fat (%)	NaCl (%)	Air (%)	Fat (%)	NaCl (%)	Air (%)	Fat (%)	NaCl (%)	Air (%)	Fat (%)	NaCl (%)	Air (%)
Second measurement cycle (repetition of first measurement): rep2	T1_1	N/A	32.00	31.70	− 8.80	32.60	31.40	31.80	32.60	32.40	32.40	30.20	33.10	33.60	30.50	31.40	32.10	29.30	33.00	33.70	29.80
	T1_2	N/A	14.90	19.00	− 31.40	16.90	17.00	18.30	18.30	16.60	16.90	17.30	17.10	17.40	17.40	16.30	17.00	16.50	17.00	17.20	16.50
	T3_1	N/A	28.70	29.70	− 8.80	29.30	29.00	30.30	31.60	29.80	29.70	29.50	30.30	30.60	29.80	29.60	29.60	28.60	29.90	30.60	29.00
	T4_1	N/A	23.40	27.50	− 20.40	25.30	26.10	25.30	26.00	25.90	25.80	24.10	26.70	27.00	24.30	25.80	25.60	23.50	26.00	27.00	23.60
	T4_2	N/A	28.00	26.70	− 6.80	29.60	28.30	32.30	29.90	29.00	28.30	28.60	30.00	29.40	28.90	28.30	29.10	27.70	29.20	30.20	28.00
	T4_3	N/A	28.20	30.70	− 11.90	28.80	28.80	29.40	30.50	29.10	29.10	28.20	30.00	30.40	28.40	28.70	29.30	27.10	29.40	30.20	27.30
	T5_1	N/A	23.60	24.90	− 19.30	25.70	24.50	25.80	26.70	24.60	24.70	24.00	25.40	26.00	24.20	24.10	25.00	23.30	25.00	26.10	23.40
	T5_2	N/A	27.00	29.40	− 14.60	28.40	28.20	28.10	31.10	28.90	27.80	27.30	28.70	29.00	27.50	28.60	28.30	26.30	29.80	29.10	26.60
	T5_3	N/A	17.20	19.50	− 25.30	19.60	18.80	21.60	21.10	18.50	18.70	19.60	19.00	19.50	19.70	18.20	18.70	18.80	18.70	19.40	19.00
	T5_4	N/A	15.20	17.80	− 28.00	17.00	16.90	20.10	19.10	16.90	17.30	17.70	17.30	17.80	17.80	16.80	16.90	17.00	16.90	18.20	17.20
	T5_5	N/A	32.20	33.10	− 3.10	32.40	31.60	34.00	33.50	31.80	31.80	30.60	33.00	33.10	31.00	31.80	32.40	30.10	32.90	32.90	30.70
	T5_6	N/A	40.30	41.60	7.10	39.10	38.00	39.60	40.20	38.70	37.10	38.50	39.70	38.20	39.10	38.90	37.60	37.60	39.50	39.20	38.20
	T5_7	N/A	21.30	23.50	− 22.60	22.60	22.50	23.90	25.30	21.90	22.10	22.50	22.80	23.20	22.60	21.60	22.00	21.40	22.10	22.90	21.60
	T6_1	N/A	25.90	28.40	− 16.50	27.10	27.00	27.40	28.20	26.60	26.60	25.40	27.20	27.30	25.50	26.00	26.50	24.50	27.00	27.10	24.70
	T6_2	N/A	26.30	29.00	− 17.80	27.20	27.70	27.10	27.50	28.40	28.70	26.60	29.60	29.70	26.90	28.00	28.50	25.80	28.90	29.00	26.00
	T6_3	N/A	37.30	37.10	2.30	36.50	35.00	37.40	37.50	36.40	35.70	35.30	37.30	36.70	35.80	35.70	35.00	34.30	36.80	36.40	34.80

^4^.

$$\lambda =\frac{{\rho }_{\text{HA}}-{\rho }_{\text{matrix material}}}{{\rho }_{\text{matrix material} }-{\rho }_{\text{collagen}}}$$

$$\mu (E)=\frac{{\alpha }_{\text{HA}}(E)\cdot {\rho }_{\text{HA}}+{\alpha }_{\text{collagen}}(E)\cdot{\rho }_{\text{collagen}}\cdot \lambda }{1+\lambda }$$
